# Metabolite profiling studies in *Saccharomyces cerevisiae*: an assisting tool to prioritize host targets for antiviral drug screening

**DOI:** 10.1186/1475-2859-8-12

**Published:** 2009-01-30

**Authors:** Konstantin Schneider, Jens Olaf Krömer, Christoph Wittmann, Isabel Alves-Rodrigues, Andreas Meyerhans, Juana Diez, Elmar Heinzle

**Affiliations:** 1 Biochemical Engineering Institute, Saarland University, 66123 Saarbrucken, Germany; 2 Institute of Virology, Saarland University, 66421 Homburg, Germany; 3 Department of Experimental and Health Sciences, University Pompeu Fabra, 08003 Barcelona, Spain; 4 Australian Institute for Bioengineering and Nanotechnology, University of Queensland, Brisbane, Australia; 5 Biochemical Engineering Institute, Technische Universität Braunschweig, Braunschweig, Germany

## Abstract

**Background:**

The cellular proteins Pat1p, Lsm1p, and Dhh1p are required for the replication of some positive-strand viruses and therefore are potential targets for new antiviral drugs. To prioritize host targets for antiviral drug screening a comparative metabolome analysis in *Saccharomyces cerevisiae *reference strain BY4742 *Matα his3Δ1 leu2Δ0 lys2Δ0 ura3Δ0 *and deletion strains *pat1Δ*, *lsm1Δ *and *dhh1Δ *was performed.

**Results:**

GC/MS analysis permitted the quantification of 47 polar metabolites and the identification of 41 of them. Metabolites with significant variation between the strains were identified using partial least squares to latent structures discriminate analysis (PLS-DA). The analysis revealed least differences of *pat1Δ *to the reference strain as characterized by Euclidian distance of normalized peak areas. The growth rate and specific production rates of ethanol and glycerol were also most similar with this strain.

**Conclusion:**

From these results we hypothesize that the human analog of yeast Pat1p is most likely the best drug target candidate.

## Background

Viruses continue to threaten human health. Well known examples are the human immunodeficiency virus (HIV) and the human hepatitis C virus (HCV) that have worldwide infected around 40 and 170 million people, respectively. While the former slowly destroys the immune system of its host resulting in acquired immunodeficiency and death due to the uncontrolled expansion of opportunistic infections, the latter, when becoming a chronic infection, leads to liver cell destruction and after decades to cirrhosis and possibly liver cell carcinoma. Even more frightening can be viruses that have a much faster disease course like some influenza strains, the SARS corona virus and hemorrhagic fever viruses which can kill their hosts in the order of days and weeks rather than several years. Without having efficient vaccines available, the best at hand to combat these threads are antiviral drugs. But there are major limitations. For many of the viruses, especially for the novel emerging viruses, there are no or only limited options for antiviral therapy. Furthermore, as most of the antivirals are specifically targeting individual viral proteins and, on the other hand, viruses most frequently exhibit high replication error rates combined with a rapid turnover of large populations, the selection of drug resistant strains within an infected host is common. While this problem can in part be solved by combining drugs with different target specificities, a setback is the increased side effects inherent to multi-drug intake [1].

The fast developing omics-technologies in the last few years have witnessed a remarkable increase in our understanding of how complex cell systems are regulated at the level of their genes, transcripts and proteins. Such types of studies are now opening up a new era in antiviral drug research [1]. Due to its genetic tractability and the new technological platforms and tools recently created, global studies in the yeast *Saccharomyces cerevisiae *have been at the forefront of such technical advances [2]. Moreover, since there is a notable gene homology between yeast and human genes and a high conservation of fundamental biochemical pathways, studies in yeast are helpful to discover fundamental cellular processes Remarkably, yeast-based studies have provided main achievements in virus research as well. These include, for example, the elucidation of key processes in viral replication and its use in antiviral drug development [2]. For drug development high throughput genomic analyses are performed to systematically screen the effect of a given drug on every gene. Such analyses will promote the identification and understanding of metabolic pathways and regulatory circuits involved in the action of a drug. This information will also provide a more detailed measure of toxicity and will therefore assist fundamentally the development of drug candidates.

Large screens for host factor requirements of different viruses have recently been completed demonstrating the intimate and complex dependence of virus expansion on cellular factors [3-6]. By targeting these so-called virus dependency factors of the host cell by drugs, virus multiplication might not only be inhibited but in addition might restrict the rapid generation of resistance. Furthermore, as groups of viruses share common replication strategies and common host factor requirements, it might become possible to even inhibit multiple viruses with a single agent [1]. For example, some of the factors required for the replication of model viruses in yeast are also required for the replication of human viruses in human cells [4,6,7] While this gives exciting future perspectives, the present challenge is how to prioritize among the many virus dependency factors experimentally determined.

BMV belongs to the family of alpha viruses which include a number of plant, animal and also human positive strand RNA viruses which exhibit common replication mechanisms. In our previous work, we have shown that the proteins Pat1p, Lsm1p-7p and Dhh1p that form a complex essentially involved in the host deadenylation-dependent mRNA decapping are required for BMV replication in yeast. Deletion of *PAT1*, *LSM1 *or *DHH1 *genes led to a comparable and dramatic inhibition of viral replication [8]. Homologues of these factors are also found in human and thus could serve as potential antiviral drug targets for human positive-strand RNA viruses such as the Hepatitis C virus [7,9-11].

Antiviral drugs should not only inhibit virus expansion but also be non-toxic for the infected cells and the host. One would expect the latter condition to be best fulfilled if the change in cellular viability characteristics would be minimal. Growth rate or even respiration are relevant primary characteristics [12]. Even more relevant are metabolic flux distributions [13] but the presumably most sensitive and most comprehensive indicator of such changes is the cellular metabolome [14]. Any modification on the level of DNA, RNA or proteins is expected to be further manifested in metabolite level changes. The scope of this work is to study the effect of the deletion of *PAT1*, *LSM1 *and *DHH1 *on central metabolites in *S. cerevisiae *and to estimate the adverse effects on the host organism and its metabolism by quantitatively comparing their metabolomes. We found that the levels of metabolites differed clearly between the reference strain *S. cerevisiae *BY4742 and the deletion strains *pat1Δ*, *lsm1Δ *and *dhh1Δ*. *PAT1 *gene deletion resulted in the smallest deviation from reference cells. This seems therefore the most promising target with respect to host cell toxicity. High throughput drug screening approaches should therefore focus on the human analogue of *PAT1*.

## Results

### Physiological growth characterization

Measured growth characteristics of studied yeast strains are summarized in Table 1. These are specific growth rate, μ, the specific glucose uptake rate, q_glucose_, the specific glycerol production rate, q_glycerol_, and the specific ethanol production rate, q_ethanol_. The specific growth rate of *pat1Δ*, 0.22 h^-1^, was quite similar to that of the reference strain, 0.28 h^-1^, whereas *dhh1Δ*, 0.16 h^-1^, and *lsm1Δ*, 0.15 h^-1^, grew clearly slower. The specific glucose uptake rates of *pat1Δ *and *dhh1Δ*, 2.00 ± 0.11 g g^-1 ^CDW h^-1 ^and 2.10 ± 0.10 g g^-1 ^CDW h^-1^, were quite similar but differed significantly compared to the reference strain, 2.45 ± 0.13 g g^-1 ^CDW h^-1^, and *lsm1Δ*, 1.67 ± 0.10 g g^-1 ^CDW h^-1^. Considering standard deviations specific glycerol production rates were similar for the reference strain and the deletion strains *pat1Δ *and *dhh1Δ *with 0.22 ± 0.03 g g^-1 ^CDW h^-1^, 0.15 ± 0.03 g g^-1 ^CDW h^-1 ^and 0.18 ± 0.02 g g^-1 ^CDW h^-1^. The specific glycerol production rate of 0.10 ± 0.02 g g^-1 ^CDW h^-1 ^for the *lsm1Δ *strain was clearly lower. The specific ethanol production rate of 1.05 ± 0.05 g g^-1 ^CDW h^-1 ^for the reference strain was higher than for the deletion strains *pat1Δ *and *dhh1Δ *with 0.72 ± 0.04 g g^-1 ^CDW h^-1 ^and 0.83 ± 0.04 g g^-1 ^CDW h^-1^. The specific ethanol production rate of the *lsm1Δ *mutant was lowest, 0.60 ± 0.03 g g^-1 ^CDW h^-1^. Based on these results the deletion of the *PAT1 *and *DHH1 *genes seem to have the least influence on the metabolism of the cell. A clear prioritization is, however not possible only considering these data. Generally, the specific uptake and production rates were proportional to the specific growth rate μ_max _as described earlier [12] which holds for the reference strain, *pat1Δ *and *lsm1Δ*. The specific rates for *dhh1Δ *were slightly higher compared to *pat1Δ*.

**Table 1:** Specific rates during growth on glucose of the reference strain *S. cerevisiae *BY4742 and the used deletion strains *pat1Δ*, *dhh1Δ *and *lsm1Δ*.

specific rate	BY4742 reference	*pat1Δ*	*dhh1Δ*	*lsm1Δ*
μ [h^-1^]	0.28 ± 0.00	0.22 ± 0.00	0.16 ± 0.01	0.15 ± 0.00
q_glucose _[g glucose/(g CDW^. ^h)]	2.45 ± 0.13	2.00 ± 0.11	2.10 ± 0.10	1.67 ± 0.10
q_glycerol _[g glycerol/(g CDW^. ^h)]	0.22 ± 0.03	0.15 ± 0.03	0.18 ± 0.02	0.10 ± 0.02
q_ethanol _[g ethanol/(g CDW^. ^h)]	1.05 ± 0.05	0.72 ± 0.04	0.83 ± 0.04	0.60 ± 0.03

μ – specific growth rate. q – specific rate.

### Metabolite identification and quantification

**Figure 1:**
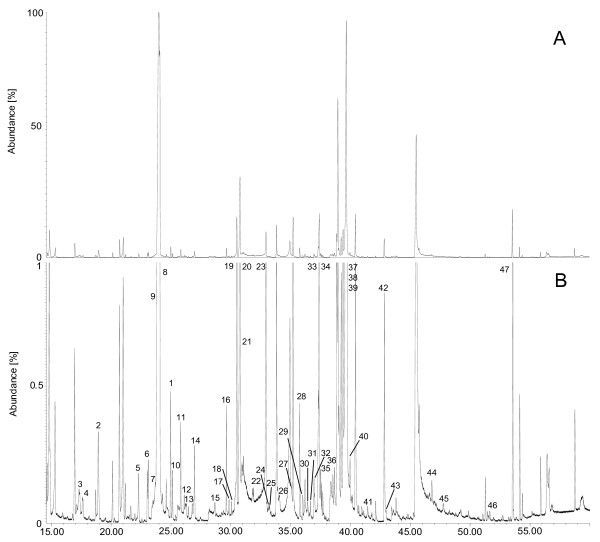
**GC/MS spectrum of a *S. cerevisiae * BY4742 *pat1Δ* cell extract.** The numerical labels of the identified compounds are specified in Table 2. (A) whole intensity rage, (B) 0 -1% of abundance range.

A typical GC/MS chromatogram of intracellular metabolites of *S. cerevisiae *BY4742 *pat1Δ *is depicted in Figure 1. This figure shows that most of the identified metabolites were in the low abundance region of the chromatogram. Most of the high abundant peaks are substances derived from column bleeding or the derivatization reagent. Of the detected 47 metabolites 41 were identified using their fragmentation pattern and retention index. Among the identified metabolites were proteinogenic and non-proteinogenic amino acids, organic acids, bases, sugars, phosphorylated sugars and several unidentified metabolites.

**Figure 2:**
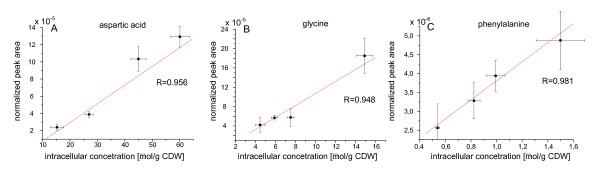
Comparison of intracellular amino acid concentrations determined by HPLC using the method of Hans *et al.* (2001) in µmol/g CDW and normalized peak areas for the amino acids (A) aspartic acid, (B) glycine and (C) phenylalanine determined by GC/MS.

Another seven metabolites could not be identified but were also included in the metabolite profiling of the three strains since they differed significantly in the investigated strains. The identified metabolites are shown in Table 2. Relative quantification was carried out using only characteristic m/z values of these identified metabolites which are also shown in Table 2. Compared to the use of the total ion current for the calculation of relative signals this resulted in a significant decrease of the signal-to-noise ratio and allowed a precise quantification even in the presence of co-eluting or incompletely separated metabolites. This kind of normalization yielded more consistent results compared to normalization to CDW or internal standards and was therefore used for the comparison of the four strains (Table 3). The resulting average error was approximately 20% comparing six samples from each strain. For checking the suitability of the chosen normalization method, intracellular amino acid concentrations of the same samples were determined by HPLC [15] and GC/MS. A good correlation was found between normalized peak areas and intracellular amino acid concentration as depicted in Figure 2 for the amino acids phenylalanine, aspartic acid and glycine.

**Table 2:** Metabolites identified in the cell extracts of BY4742 and the used deletion mutants using the software AMDIS v2.0 and a TMS library.

metabolite	peak number	m/z	#TMS/MeOx
glycine	1	102	2
alanine	2	116	2
pyruvic acid	3	174	1/MeOx
lactic acid	4	117	2
valine	5	144	2
u. m. #1	6	110	n.k.^1^
ethanolamine	7	174	2
glycerol	8	218	3
isoleucine/leucine	9	158	2
succinic acid	10	247	2
uracil	11	241	2
fumaric acid	12	245	2
serine	13	204	3
threonine	14	218	3
homoserine	15	218	3
malic aicd	16	233	3
u. m. #2	17	188	n.k.^1^
erythritol	18	217	4
aspartic acid	19	218	3
u. m. #6	20	258	n.k.^1^
cytosine	21	254	2
α-ketoglutaric acid	22	198	2/MeOx
glutamic acid	23	246	3
phenylalanine	24	192	2
ribose	25	217	4/MeOx
asparagine	26	231	3
2-aminoadipic acid	27	260	3
orotic acid	28	254	3
glycerol-1-phosphate	29	370	4
glutamine	30	156	3
N-acetyl-glutamic acid	31	216	2
u. m. #3	32	257	n.k.^1^
citric acid	33	273	4
ornithine	34	258	4
arginine	35	256	5
adenine	36	279	2
lysine	37	230	4
histidine	38	356	3
manitol	39	307	6
tyrosine	40	218	3
u. m. #4	41	258	n.k.^1^
inositol	42	305	6
xylulose-5-phosphate	43	315	5/MeOx
glucose-6-phosphate	44	387	6/MeOx
u. m. #7	45	387	n.k.^1^
u. m. #5	46	204	n.k.^1^
trehalose	47	361	8

Peak numbers correspond to the labelling of the data points in Figure 2. #TMS denotes tert-methyl-silyl residues in the detected analyte originating from derivatization, MeOx denotes methoxymation of the metabolite. ^1^n.k., not known; u.m., unknown metabolite.

**Table 3:** Peak areas of detected metabolites normalized to total peak area in BY4742 and the three deletion strains *pat1Δ*, *dhh1Δ *and *lsm1Δ *using GC/MS.

	BY4742		*pat1Δ*		*dhh1Δ*		*lsm1Δ*	
metabolite	mean	s. d.	mean	s. d.	mean	s. d.	mean	s. d.
glycine	4.67E-05	1.10E-05	6.07E-05	1.12E-05	5.68E-05	4.58E-06	1.97E-04	2.26E-05
alanine	1.28E-04	2.68E-05	1.34E-04	3.07E-05	2.08E-04	3.27E-05	1.06E-03	1.71E-04
pyruvic acid	1.02E-05	5.52E-06	9.87E-06	2.62E-06	6.24E-06	8.64E-07	4.59E-06	6.12E-07
lactic acid	1.65E-05	1.72E-06	1.22E-05	2.44E-06	7.95E-06	1.47E-06	7.67E-06	2.35E-06
valine^E^	4.73E-05	8.82E-06	6.02E-05	9.70E-06	4.91E-05	7.67E-06	1.80E-04	2.54E-05
u. m. #1	2.25E-05	5.10E-06	3.33E-05	6.92E-06	4.38E-05	1.92E-05	9.97E-06	2.39E-06
ethanolamine	8.47E-06	3.27E-06	6.76E-06	1.37E-06	1.14E-05	1.74E-06	2.11E-05	6.15E-06
glycerol	3.38E-04	5.49E-05	3.36E-04	2.36E-05	6.98E-04	6.75E-05	5.96E-04	1.01E-04
isoleucine/leucine^E^	5.32E-06	2.21E-06	5.08E-06	1.04E-06	7.69E-06	5.53E-07	2.22E-05	3.13E-06
succinic acid	4.71E-06	1.74E-06	2.59E-06	1.08E-07	4.65E-06	2.64E-07	1.01E-05	1.09E-06
uracil	1.55E-05	3.05E-06	1.72E-05	3.08E-06	1.70E-05	5.16E-06	1.99E-05	2.05E-06
fumaric acid	4.56E-06	1.43E-06	6.06E-06	9.78E-07	8.08E-06	3.39E-07	1.78E-05	2.32E-06
serine	2.17E-05	5.77E-06	2.10E-05	2.57E-06	3.94E-05	3.32E-06	3.45E-05	6.43E-06
threonine^E^	1.94E-05	4.14E-06	2.19E-05	2.94E-06	3.70E-05	3.35E-06	5.99E-05	5.37E-06
homoserine^N^	9.32E-06	1.60E-06	9.77E-06	1.52E-06	1.71E-05	1.15E-06	2.50E-05	2.18E-06
malic acid	3.98E-06	9.76E-07	7.88E-06	9.28E-07	1.23E-05	8.03E-07	2.30E-05	3.55E-06
u. m. #2	2.41E-06	3.99E-07	2.50E-06	4.32E-07	3.69E-06	2.40E-07	4.60E-06	4.01E-07
Erythritol^N^	2.13E-06	2.67E-07	1.75E-06	2.73E-07	n.d.^1^	n.d.^1^	n.d.^1^	n.d.^1^
aspartate	2.39E-05	3.44E-06	3.89E-05	3.18E-06	1.29E-04	1.20E-05	1.03E-04	1.43E-05
u. m. #6	7.99E-05	1.28E-05	7.55E-05	1.50E-05	4.56E-05	2.41E-06	4.82E-05	9.02E-06
cytosine	7.49E-06	1.03E-06	6.97E-06	1.21E-06	5.64E-06	1.90E-06	5.94E-06	1.05E-06
α-ketoglutaric acid	3.89E-07	1.84E-07	6.66E-07	9.73E-08	4.56E-07	8.42E-08	n.d.^1^	n.d.^1^
glutamate	6.97E-05	1.84E-05	1.45E-04	1.84E-05	1.33E-04	2.12E-05	8.20E-05	1.03E-05
phenylalanine^E^	2.56E-06	6.33E-07	3.94E-06	4.11E-07	4.88E-06	7.75E-07	3.28E-06	4.78E-07
ribose	1.50E-06	4.90E-07	8.37E-07	1.52E-07	4.72E-07	7.98E-08	5.02E-07	1.66E-07
asparagine	1.79E-06	6.49E-07	2.34E-06	3.99E-07	3.91E-06	1.09E-06	2.37E-06	7.65E-07
2-aminoadipic acid	4.03E-06	1.22E-06	2.32E-06	3.72E-07	8.49E-07	1.57E-07	4.12E-06	4.93E-07
orotic acid	6.56E-05	2.11E-05	3.38E-05	2.74E-06	1.55E-05	2.70E-06	7.66E-05	1.05E-05
glycerol-1-phosphate	5.65E-07	1.05E-07	6.80E-07	4.76E-08	5.17E-07	1.10E-07	n.d.^1^	n.d.^1^
glutamine	1.86E-06	5.48E-07	4.54E-06	3.26E-06	1.46E-05	4.06E-06	4.19E-06	4.66E-06
N-acetyl-glutamic acid	5.62E-07	1.34E-07	1.63E-06	3.29E-07	5.72E-07	9.51E-08	n.d.^1^	n.d.^1^
u. m. #3	1.06E-05	7.65E-06	8.19E-06	1.98E-06	1.88E-06	1.75E-07	1.31E-05	1.94E-06
citric acid	9.43E-06	1.82E-06	1.47E-05	1.51E-06	4.79E-04	7.40E-05	1.28E-05	3.12E-06
ornithine	3.69E-06	1.17E-06	1.17E-05	1.40E-06	1.40E-05	2.94E-06	1.69E-06	3.42E-07
arginine	4.17E-06	1.10E-06	1.19E-05	1.88E-06	6.33E-06	1.33E-06	8.16E-07	4.37E-07
adenine	3.78E-06	8.22E-07	3.84E-06	9.41E-07	3.86E-06	1.24E-06	4.82E-06	7.76E-07
lysine^E^	1.52E-04	4.67E-05	2.16E-04	1.11E-05	4.15E-04	3.30E-05	4.44E-04	5.42E-05
histidin^E^e	1.36E-05	1.63E-06	1.93E-05	8.38E-06	2.83E-05	3.25E-06	1.23E-05	8.86E-07
mannitol^N^	4.96E-07	7.11E-08	1.03E-06	1.34E-07	6.30E-07	9.23E-08	9.67E-07	1.55E-07
tyrosine^E^	8.22E-06	3.02E-06	2.14E-05	1.96E-06	1.93E-05	1.76E-06	1.72E-05	1.35E-06
u. m. #4	1.74E-06	3.53E-07	8.75E-07	1.18E-07	9.58E-07	1.96E-08	1.15E-06	2.03E-07
inositol	4.81E-05	9.72E-06	4.23E-05	3.34E-06	3.28E-05	1.83E-06	3.41E-05	3.38E-06
xylulose-5-phosphate	3.15E-06	1.36E-06	1.12E-06	1.55E-07	1.22E-06	2.81E-07	7.68E-07	6.53E-08
glucose-6-phosphate	6.96E-06	1.59E-06	5.92E-06	1.56E-06	2.11E-06	4.92E-07	1.54E-06	1.15E-06
u. m. #7	3.44E-06	5.64E-07	1.70E-06	6.00E-07	3.39E-06	1.12E-06	4.58E-06	5.05E-07
u. m. #5	2.56E-06	3.60E-07	2.73E-06	4.09E-07	1.69E-06	2.72E-07	1.75E-06	5.30E-07
trehalose^N^	5.08E-06	9.10E-07	1.87E-04	5.06E-05	9.54E-05	2.70E-05	9.61E-04	2.13E-04

^E ^indicates essential amino acids; ^N ^indicates metabolites usually not observed in human cells.

### Strain comparison using GC/MS

**Figure 3:**
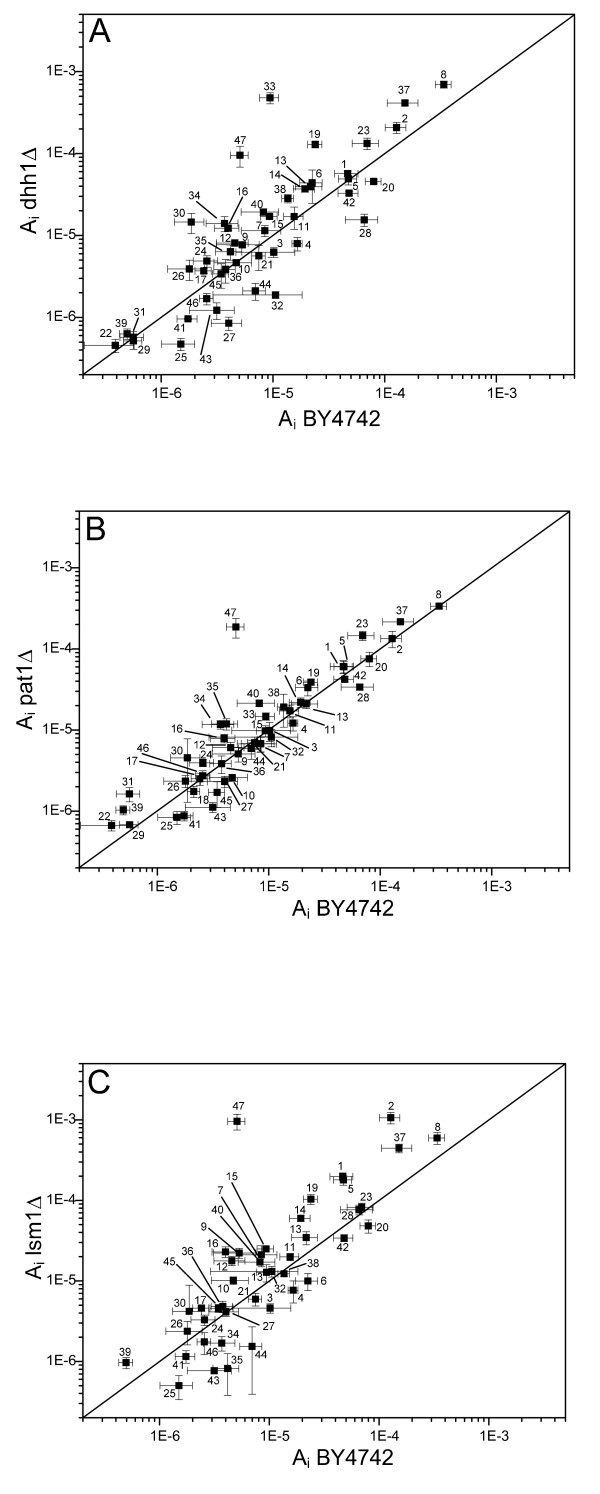
**Comparison of intracellular metabolites of *S. cerevisiae* strains analyzed by GC/MS using normalized peak areas, I_i_.** (A) *dhh1Δ* mutant versus reference BY4742, (B) *pat1Δ* versus reference BY4742, (C) *lsm1Δ* versus reference BY4742. The solid line indicates identical concentrations in both strains. Data points denote the mean of the normalized areas taken from six independent samples. Error bars in both directions indicate the corresponding standard deviations. The numerical labels of the single metabolites are defined in Table 2.

The three deletion strains *pat1Δ, dhh1Δ *and *lsm1Δ *and the reference strain BY4742 were compared using relative pool sizes of intracellular metabolites, characterized by normalized peak areas, derived from GC/MS measurements. Figure 3 compares the intracellular metabolite pool sizes in the three deletion strains to the reference strain BY4742. Metabolites on the straight line have the same concentration in both strains.

**Figure 4:**
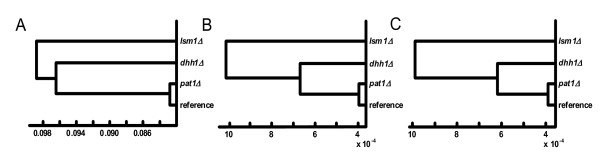
**Strain comparison based on intracellular metabolites extracted form *S. cerevisiae* BY4742 and the tree deletion strains pat1Δ, dhh1Δ and lsm1Δ and analyzed using GC/MS.** A – full data set; B – data without metabolites not observed in human as indicated in Table 3; C – same as B but with additional removal of essential amino acids that are not synthesized in human. The distances between the four strains correspond to the Euclidian distance calculated by using the normalized areas of the extracted metabolites of the four strains.

Based on the normalized peak areas, the Euclidian distance between the three deletion strains and the reference strain was calculated and used for characterizing similarity. In this way we could estimate the influences of the deletions of the *PAT1*, *DHH1 *and *LSM1 *genes on the cellular metabolism. The comparison was made in three steps and is presented in dendrograms (Figure 4). In a first calculation the whole data set was used (Figure 4A). In a second all metabolites usually not observed in human that are indicated in Table 3 were used (Figure 4B). Finally, also the essential amino acids that are not synthesized by human were removed in a third analysis (Figure 4C). In all cases it is evident that the deletion of the *PAT1 *gene caused the smallest differences in the metabolite pattern compared to the reference strain. Therefore, the whole data set was used for further analysis. The deletion of the genes *DHH1 *and *LSM1 *caused larger deviations. This is in accordance with the specific growth rates of the four strains describing the overall fitness of the cells.

### Principal component analysis and strain separation

**Figure 5:**
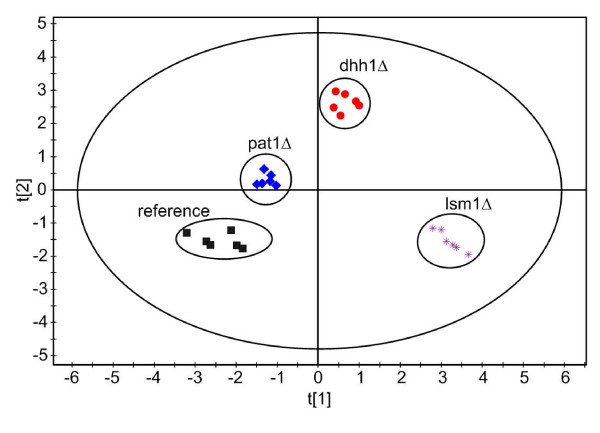
**PCA scores plot based on normalised peak areas from GC/MS analysis of cell extracts and the four strains BY4742 (reference), pat1Δ, dhh1Δ and lsm1Δ (each one n=6).** Analysis was conducted using SIMCA-P+ 11.5 (Umetrics, Malmö, Sweden). Data were pareto scaled and the first two components were autofitted.

Principal component analysis (PCA) using normalized peak areas from GC/MS analysis of the cell extracts reveals clear separation of the four strains (WT, *pat1Δ, lsm1Δ *and *dhh1Δ*). Figure 5 shows the corresponding scores plot. The four strains form distinct clusters using two parallel cultures and three sampling times each. These results confirm that cells were in true exponential growth and therefore constant concentrations of intracellular metabolites could be assumed during the entire sampling period. At the different sampling times biomass concentration was varying between 0.73 – 1.44 g CDW l^-1^, but did obviously not cause significant disturbances. These results also indicate the applicability of the chosen normalization procedure since resulting PLS scores are randomly distributed within their specific cluster. Figure 5 again shows that metabolites measured in the *pat1Δ *deletion strain are least deviating from those in the reference strain. The two deletion strains *lsm1Δ *and *dhh1Δ *are obviously more distant agreeing with the Euclidian distance calculations depicted in Figure 4.

### Identification of statistically significant metabolites using PLS-DA

**Figure 6:**
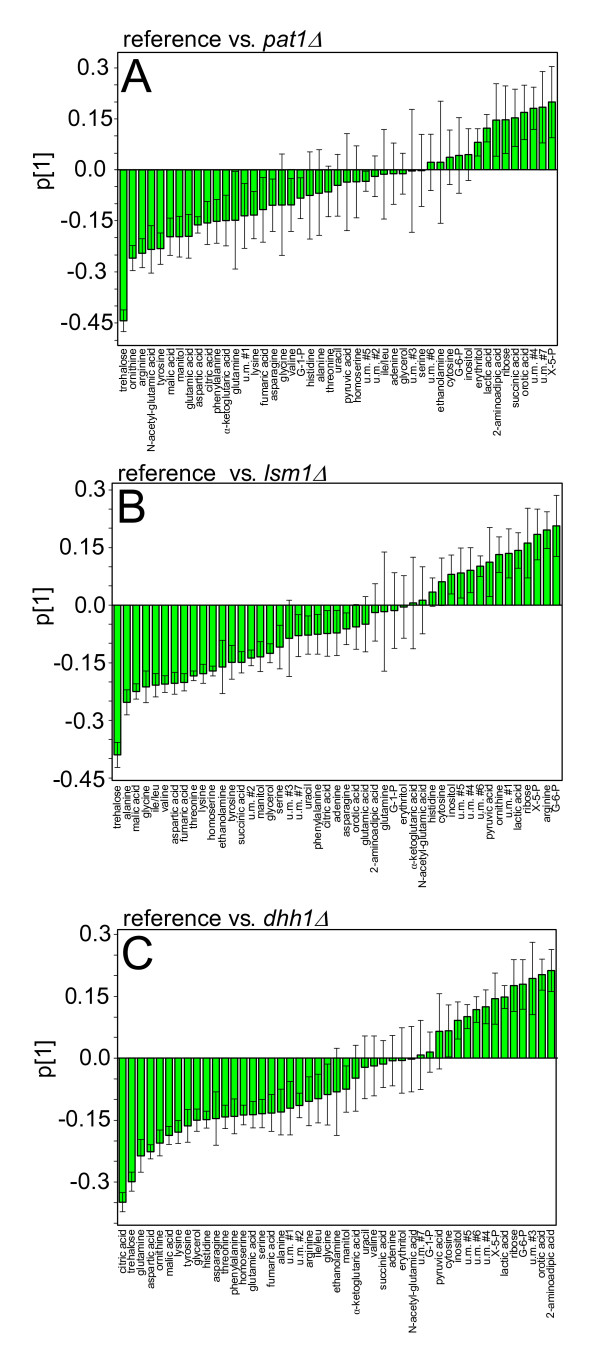
**Loading plots of PLS-DA analysis of (A) reference and pat1Δ, (B) reference and lsm1Δ and (C) reference and dhh1Δ (each one n=6) cell extracts from GC/MS analysis using normalized peak areas.** Error-bars indicate jack-knifed confidence intervals. Values below zero indicate a higher concentration compared to the reference strain. Values above zero denote lower concentrations compared to the reference strain. Data were pareto scaled and analysis was carried out using SIMCA-P+ 11.5 (Umetrics, Malmö, Sweden).

**Figure 7:**
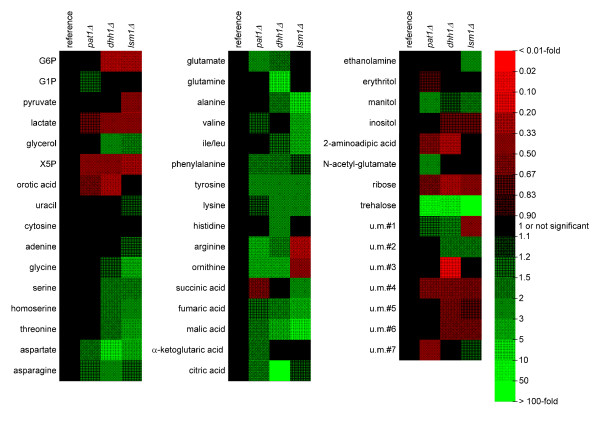
**Heat map of 47 metabolites that show statistically significant changes between the reference strain (n=6) and the three deletion strains pat1Δ, dhh1Δ and lsm1Δ (each one n=6).** Red color denotes lower concentrations in the deletion strains; green color indicates higher concentrations in the deletion strains. Statistically significant metabolites were identified using the loading plots shown in Figure 6. G6P, glucose-6-phosphate; G1P, glycerol-1- phosphate; X5P, xylulose-5-phosphate; u. m.; unknown metabolite; WT, reference strain BY4742.

The selection of statistically significant metabolites was made using loading plots for each of the three comparisons (Figure 6). All metabolites whose confidence intervals do not include zero were assumed statistically significant. Strain comparison was carried out using only these metabolites. The concentration differences are presented in a heat plot (Figure 7).

The most significant differences between the reference strain BY4742 and the three deletion strains was the accumulation of intracellular trehalose. Intracellular pools were clearly increased in the three deletion strains. The highest concentration was found in the *lsm1Δ *strain (200 times the concentration in the reference strain) while the intracellular pools of the two strains *pat1Δ *and *dhh1Δ *were approximately 25 times higher compared to the reference strain pool size of intracellular trehalose. Increased intracellular trehalose levels were observed when applying a heat shock or oxidative stress to the cells [16]. The measured TCA cycle metabolites succinic acid, malic acid, fumaric acid, α-ketoglutaric acid and citric acid were more accumulated in the deletion strains. Especially malic acid was concentrated 2 times, 3 times and 6 times higher in the strains *pat1Δ, dhh1Δ *and *lsm1Δ *compared to the reference strain. The citric acid pool size of the deletion strain *dhh1Δ *was 5 times higher than the corresponding reference strain pool size. The urea cycle metabolites ornithine and arginine were slightly higher concentrated in the deletion strains *pat1Δ *and *dhh1Δ *but levels are decreased in the *lsm1Δ *strain. Fumaric acid, which is also produced via the urea cycle, showed a higher abundance in the *lsm1 *deletion strain compared to the reference strain and *pat1Δ *and *dhh1Δ *indicating that the urea cycle was affected in another way in this strain.

Intracellular amino acid concentrations were always higher in the deletion strains or not significantly changed. The only exception was arginine in the *lsm1 *deletion strain as discussed above. A comparison of the reference strain *S. cerevisiae *BY4742 with the non-auxotrophic wild type *S. cerevisiae *ATCC 32167 revealed that the intracellular concentrations of leucine, histidine and lysine were always higher than in the non-auxotrophic wild type strain. Here the concentration of leucine, histidine and lysine of strain BY4742 were 13.1, 39.2 and 93.9 μmol/g CDW. In the wild type strain *S. cerevisiae *ATCC 32167 all other intracellular amino acid concentrations were in the same range as in *S. cerevisiae *BY4742, except the three amino acids supplied in the medium with concentrations 0.9, 3.4 and 9.5 μmol/g CDW, respectively. Their concentrations were approximately 10 times higher compared to *S. cerevisiae *ATCC 32167. This difference can be explained by the feeding of these essential amino acids in excess to the *S. cerevisiae *BY4742 and mutant cultures.

## Discussion

Previously it was shown that the proteins Pat1p, Lsm1p-7p and Dhh1p are required for BMV replication in yeast [8]. Homologues of these factors are also found in humans and can serve as potential antiviral drug targets for human positive strand RNA viruses like Hepatitis C virus [7,9-11]. To analyze which of the proteins is the most promising target for a new inhibitory drug, it is of primary importance to show the efficacy of the target inhibition but at the same time it is equally important to find out which of the potential targets would be least harmful to the host organism. Such studies are difficult in human cells and yeast seems suitable as model organism to prioritize amongst the potential targets. It is not a priori clear which parameter would be most reasonable to measure deletion effects most sensitively. We think however that metabolome analysis is a most ideal candidate to describe the phenotype of a cell [17-19] since it reflects effects on the gene, RNA and protein levels comprehensively.

All three genes, *PAT1*, *LSM1 *and *DHH1 *essential for virus propagation are dispensable for yeast growth. However, the quantitative effects of the individual deletions on physiological parameters as growth and substrate consumption rates and intracellular metabolite levels were different. In our study we could identify 41 intracellular metabolites and 6 metabolites of unknown chemical structure in polar extracts of *S. cerevisiae *using GC/MS covering nearly all parts of central metabolism. The average variation of intracellular metabolite concentrations of the six independent samples of one single strain was around 20%. The variation in the analysis of polar leaf extracts of *Arabidopsis thaliana *resulted in 101 identified metabolites and 113 of unknown chemical structure and a biological variation of 35% in average [20]. Strelkov *et al*. [21] identified 88 intracellular metabolites in the polar extracts of *Corynebacterium glutamicum*. More metabolites could be identified with less stringent identification criteria; however here we used the standard identification criteria of the AMDIS software. As Figure 2 shows, there are a lot more compounds visible in the chromatogram than identified and used for strain comparison in this work. Using less stringent identification rules resulted in the identification of 39 additional metabolites which were, however, not used for strain comparison. Most importantly, this study showed clear differences on the intracellular metabolome changes between the different gene deletion strains. Intracellular metabolite levels of all three deletion strains, *pat1Δ*, *lsm1Δ *and *dhh1Δ *differed from those of the reference strain *S. cerevisiae *BY4742. It was, however, found that the deletion of *PAT1 *showed the least differences to the reference strain. Some of the metabolites found (Table 3) are usually not observed in human, others are essential amino acids that are not synthesized by human. It is interesting to see that metabolome differences were similar with and without these metabolites. This indicates a certain robustness of the method. The most pronounced non-human metabolite, trehalose, may, however, be replaced by glycogen in human. The other non-human metabolites, erythritol, mannitol and homoserine are changed only little. Intracellular concentrations of amino acids essential in human are changed significantly in yeast but this did not have any significant effect on the overall analysis. The metabolome results are supported by the fact that the measured specific growth rates of the *pat1Δ *strain and the reference strain were most similar. In general it can however be expected that metabolome analysis is much more sensitive than mere growth rate determination and will, together with genomic, transcriptomics and proteomic assays, greatly enhance our understanding of mechanisms of drug effect and of adverse drug reactions [14].

## Conclusion

In this work the intracellular metabolome of a series of *S. cerevisiae *strains with deletions of genes coding for the cellular proteins Pat1p, Lsm1p, and Dhh1p was analyzed. These proteins are required for the replication of some positive-strand viruses and are therefore potential targets for new antiviral drugs. In our attempt to prioritize host targets for antiviral drug screening we arrived at the conclusion that the human gene corresponding to Pat1p seems the most promising therapeutic target of the selected proteins essential for the development of antiviral drugs against positive-strand RNA viruses. The results presented here also show that intracellular metabolome analysis is a sensitive and powerful tool to tackle problems of this type.

## Methods

### Organisms and cultivation

*S. cerevisiae *ATCC 32167 was purchased from ATCC. *S. cerevisiae *deletion mutants with the parental phenotype BY4742 *Matα his3Δ1 leu2Δ0 lys2Δ0 ura3Δ0 *were obtained from Open Biosystems (Heidelberg, Germany). The derived yeast deletion mutants *Δlsm1 Δpat1 *and *Δdhh1 *are resistant to the antibiotic geneticin and exhibit auxothrophies for histidine, leucine, lysine and uracil. Yeast cells were grown on YPD-agar plates (10 g/l yeast extract, 20 g/l peptone, 20 g/l glucose) containing 200 mg/l Geneticin. Cultivation was carried out first on complex YPD-medium at 30°C followed by a second pre-culture on defined synthetic medium containing (NH)_4_HPO_4 _1 g/l, (NH)_4_SO_4 _8.75 g/l, MgSO_4_·7 H_2_O 1 g/l, citric acid 1.1 g/l, CaCl_2_·2 H_2_O 0.15 g/l, glucose 20 g/l, 0.5 M Na-phosphate buffer (pH 6.0) 100 ml/l, 100× trace element solution 10 ml/l and 100× vitamin solution 10 ml/l. Lysine, leucine (each 120 mg/l), histidine and uracil (each 80 mg/l) were added from 100× stock solutions to allow growth of the deletion mutants. 100× vitamin solution consists of myo-inositol 301.5 mg/l, Ca-panthotenate 150 mg/l, thiamin/HCl 30 mg/l, pyridoxine/HCl 7,5 mg/l and biotin 0,15 mg/l. 100× trace element solution consists of FeCl_3_·6 H_2_O 75 mg/l, MnSO_4_·H_2_O 53 mg/l, ZnSO_4_·7 H_2_O 45 mg/l and CuSO_4_·5 H_2_O 12 mg/l [12]. The main culture was carried out in defined synthetic medium using 1000 ml baffled shake flasks. The same medium without additional amino acids and uracil was also used for the cultivation of *S. cerevisiae *ATCC 32167 that is the wild type of the used reference strain BY4742.

For inter-strain comparison of intracellular metabolite concentrations, two parallel cultures were grown, and three samples were taken during the exponential growth phase. Cells were harvested at optical densities between 2 and 3. Exponential growth was observed for at least 30 min after taking samples for metabolite extraction to ensure that no changes in cell metabolism occurred, due to substrate limitation effects.

### Analytics

Cell dry weight (CDW) was determined gravimetrically after washing cells twice with double distilled water. After centrifugation (10 min, 8,000 × g, 4°C, Biofuge stratos, Heraeus, Hanau, Germany) the drying was performed at 80°C until constant weight was observed. Optical density (OD_660 nm_) was determined at 660 nm (Novaspec II, Pharmacia Biotech, Freiburg, Germany). The linear correlation between CDW and OD_660 _is CDW [g/l] = 0.51 [g/l]·OD_660 nm_. OD-CDW correlations of the four strains were identical.

Concentrations of extracellular glycerol, glucose and ethanol were determined by HPLC (Kroma System, Kontron Instruments, Neufahrn, Germany) with an Aminex HPX-87H column (300 × 7,8 mm; Bio-Rad, Hercules, USA) and an isocratic flow of 0.5 ml/min 22 mM H_2_SO_4 _at 35°C. Glucose and ethanol concentrations in the supernatant were determined using enzymatic kits (Boehringer Mannheim, R-Biopharm GmbH, Darmstadt, Germany) in addition to HPLC analysis. Intracellular amino acid concentrations were determined by HPLC using an OPA method [22].

### Quenching and metabolite extraction

For metabolome analysis three independent samples of 10 ml were drawn from each of the two cultures during the exponential growth phase and transferred immediately under vigorous shaking to a 50 ml falcon tube, filled with pre-cooled quenching solution (-40°C, 60% methanol, 10 mM HEPES, pH 7.5). Separation of cells and medium was performed by centrifugation (5 min, 8,000 × g, -19°C, Biofuge stratos, Heraeus, Hanau, Germany) [23]. The temperature was always below -25°C during the whole quenching process. After an additional washing step the metabolite extraction was achieved by incubating the pellet with 2 ml of boiling water for 15 min. Cell-debris was separated by centrifugation (10 min, 1,500 × g, 4°C, Labofuge 400R, Heraeus, Hanau, Germany) and extracted again with 750 μl chloroform (37°C, 10 min). The two phases were combined and separated again by centrifugation. The polar phase was further analyzed. The sampling process until quenching was not oxygen limited. This was ensured by measuring dissolved oxygen (DO) concentration using an optical method [24]. DO concentrations were always above 30% of air saturation.

### GC/MS analysis of intracellular metabolites

Prior to GC/MS analysis 100 μl of the cell extract was lyophilized (Lyovac GT2, GEA Lyophil GmbH, Huerth, Germany). The remaining powder was derivatised using 25 μl 20 g/l methoxylamine/HCl in pyridine for oximation (30 min, 80°C) followed by 50 μl N-methyl-N-trimethylsilyl-trifluoroacetamide (MSTFA) for silylation. Sample analysis was carried out on a HP6890 GC System using a Mass Selective Detector 5973 (Agilent Technologies, Waldbronn, Germany). Full scan mass spectra were acquired ranging from 30 to 600 amu using a scan rate of 9 scans/s. Separation was performed using a 60 m × 0.2 mm I.D. fused silica HP-5 ms column (Agilent Technologies, Waldbronn, Germany) and a helium carrier gas flow rate of 0.7 ml/min. After 1 min at 70°C the temperature was increased to 75°C (1°C/min) followed by a second increase to 315°C (5°C/min) and finally to 340°C (25°C/min). 1μl of derivatised sample was injected splitless for 2 min using a PTV with a temperature gradient from 75°C to 340°C with a rate of 360°C/min. injector. Temperatures of ion source and transfer liner were 200°C and 240°C. For electron impact ion generation a 70-eV electron beam was used. Intracellular amino acid concentrations were determined by HPLC using the OPA method [15].

### Metabolite identification and deconvolution

Metabolite identification and signal deconvolution was performed using the AMDIS software (version 2.64, National Institute of Standards and Technology, Gaithersburg, USA) as well as the Enhanced ChemStation software (Agilent Technologies, Waldbronn, Germany). Identification of metabolites is based on the comparison of measured mass spectra and retention indices (RI) with in-house library entries [25] of pure component mass spectra and retention indices or the National Institute of Standards and Technology (NIST) library. Retention indices were scaled using a standard of 33 hydrocarbons ranging from C_8 _to C_40_. To determine the differences among the reference and the deletion strains a relative quantification is sufficient. Relative quantification of metabolites was carried out using peak areas of characteristic m/z ratio intensities of these metabolites that were normalized to the integral of the total intensity over the whole chromatogram.

### PLS modeling and PCA

To find statistically significant differences between the metabolite profiles of the reference strain and the three deletion strains, the partial least squares to latent structures (PLS) regression method was applied [26]. This is commonly used in the analysis of data from multivariate studies to find significant relationships between different classes, here the reference strain and deletion strains. The PLS estimation resulted in three sets of model parameters each one comparing one of the deletion strains with the reference strain. Calculation of the model parameters was carried out using SIMCA-P 11.5+ (Umetrics, Malmö, Sweden). To reduce the impact of artifacts and noise during the modeling process, pareto scaling was used for all variables. For data visualization and selection of interesting metabolites loading plots with jack-knifed confidence intervals were chosen as described by Wiklund *et al*. [27].

### Euclidian distance

Euclidian distances between different strains were calculated using Matlab (version 7.2.0.232, The Math Works, Inc., Boston, USA). The Euclidian distance d(x, y) between two points x = (x_1_, x_2_,..., x_n_) and y = (y_1_, y_2_,..., y_n_) in a n-dimensional space is defined as

$$d\left(x,y\right)=\sqrt{\sum_{i=1}^{n}{\left(x_{i}-y_{i}\right)}^{2}}.$$

For each knock-out strain the Euclidian distance to the reference strain was calculated and visualized in the form of a dendrogram. Each vector includes the normalized areas of the corresponding metabolites which are statistically significant based on the PLS-DA (PLS-discriminant analysis).

### Chemicals

Peptone was obtained form Bacto (Sparks, USA). Yeast Extract and nutrient agar were obtained from Difco (Sparks, USA). MSTFA was obtained form Macherey-Nagel (Düren, Germany). All other chemicals used were of analytical grade and obtained either from Sigma-Aldrich (St. Louis, USA) or Fluka (Buchs, Switzerland).

## Competing interests

The authors declare that they have no competing interests.

## Authors' contributions

KS was involved in conception and design, made all experiments and most of the data evaluation and interpretation, drafted major parts of the manuscript and revised the manuscript. JOK contributed during the set-up of the GC-MS-PTV system and was involved in the initial experiments. CW was involved in conception and design and contributed with his know how for metabolome analysis using GC-MS. IAR was involved in conception and design and in manuscript revision. AM and JD were involved in conception and design, drafting of the manuscript, data interpretation and in manuscript revision. EH was the primary responsible for the whole work, was involved in conception and design, drafting of the manuscript, data analysis and interpretation and in manuscript revision. All authors read and approved the final manuscript.
